# Supplementary material for: The Inability to Disassemble Rad51 Nucleoprotein Filaments Leads to Aberrant Mitosis and Cell Death
Source: Biomedicines. 2023 May 15;11(5):1450. doi: 10.3390/biomedicines11051450 (PMC10216663; doi:10.3390/biomedicines11051450)
Supplement: Supplementary file 1 [file biomedicines-11-01450-s001.zip › biomedicines-2363296-supplementary.pdf]

Table S1. *Saccharomyces cerevisiae* A364a strains used in the study

**Strain number**	**Genotype**	**Origin/References**
NK1	*MATa ura3-52 trp1-289 leu2-3,112 bar1::LEU2*	[118]
NK81	*MATa ura3-52 trp1-289 leu2-3,112 bar1::LEU2 rad52::TRP1*	NK1 *rad52::TRP1*
NK219	*MATα ura3-52 trp1-289 leu2-3,112 bar1::LEU2*	[119]
NK1325	*MATa-inc trp1-289 ura3::NAT leu2::LEU2-Pgal-HO HEM13::HOsite-URA3 pif1-m2-TRP1-pif1-m1*	[119]
NK4691<br>NK4692<br>NK4693	*MATa-inc trp1-289 leu2::PGAL1-HO-LEU2 ura3-52::KAN-HO site-URA3*	[46]
NK4805<br>NK4806<br>NK4807<br>NK4808	*MATa-inc trp1-289 leu2::PGAL1-HO-LEU2 ura3-52::KAN-HO site-URA3 srs2::TRP1*	[46]
NK5854<br>NK5855	*MATa-inc trp1-289 leu2::PGAL1-HO-LEU2 ura3-52::KAN-HO site-URA3 srs2:HYG*	NK4805 *srs2::HYG*
NK5856<br>NK5857		NK4807 *srs2::HYG*
NK5858<br>NK5859	*MATa-inc trp1-289 leu2::PGAL1-HO-LEU2 ura3-52::KAN-HO site-URA3 rad51::TRP1-PGAL1-RAD51*	NK4691 *rad51::TRP1-PGAL1-RAD51*
NK5860		NK4692 *rad51::TRP1-PGAL1-RAD51*
NK5861<br>NK5862	*MATa-inc trp1-289 leu2::PGAL1-HO-LEU2 ura3-52::KAN-HO site-URA3 rad51::TRP1-PGAL1-RAD51 srs2::HYG*	NK5854 *rad51::TRP1-PGAL1-RAD51*
NK5863		NK5856 *rad51::TRP1-PGAL1-RAD51*
NK5864<br>NK5865	*MATa-inc trp1-289 leu2::PGAL1-HO-LEU2 ura3-52::KAN-HO site-URA3 rad51::TRP1-PGAL1-RAD51 rad54::NAT*	NK5858 *rad54::NAT*
NK5866		NK5860 *rad54::NAT*
NK5868<br>NK5869	*MATa-inc trp1-289 leu2::PGAL1-HO-LEU2 ura3-52::KAN-HO site-URA3 rad51::TRP1-PGAL1-RAD51 srs2::HYG rad54::NAT*	NK5861 *rad54::NAT*
NK5870<br>NK5871		NK5863 *rad54::NAT*
NK5895<br>NK5896<br>NK5897	*MATa ura3-52 trp1-289 leu2-3,112 bar1::LEU2 rad54::NAT*	NK1 *rad54::NAT*
NK5898<br>NK5899<br>NK5900	*MATα ura3-52 trp1-289 leu2-3,112 bar1::LEU2 srs2::srs2(aa1-860)*	NK219 *srs2::srs2(aa1-860)*
NK5901	*MATa/α ura3-52/ura3-52 trp1-289/trp1-289 leu2-3,112/leu2-3,112 bar1::LEU2/bar1::LEU2 SRS2/ srs2::srs2(aa1-860) rad54::NAT/RAD54*	NK5895 x NK5898
NK5902		NK5896 x NK5899
NK5903		NK5897 x NK5900
NK5949	*MATα ura3-52 trp1-289 leu2-3,112 bar1::LEU2 rad54::NAT*	NK5902 sporulation
NK5951	*MATa ura3-52 trp1-289 leu2-3,112 bar1::LEU2 srs2::HYG*	NK1 *srs2::HYG*

NK5953	*MATa/α ura3-52/ura3-52 trp1-289/trp1-289 leu2-3,112/leu2-3,112 bar1::LEU2/bar1::LEU2 SRS2/srs2::HYG rad54::NAT/RAD54*	NK5949 x NK5951
NK6390	*MATa-inc trp1-289 leu2::P$_{GAL1}$-HO-LEU2 ura3-52::KAN-HO site-URA3 rad54::NAT*	4691 *rad54::NAT*
NK6391<br>NK6392		4692 *rad54::NAT*
NK6724	*MATa-inc trp1-289 leu2::P$_{GAL1}$-HO-LEU2 ura3-52::KAN-HO site-URA3 rad55::TRP1-P$_{GAL1}$-RAD55*	4691 *rad55::TRP1-P$_{GAL1}$-RAD55*
NK6725<br>NK6726		4692 *rad55::TRP1-P$_{GAL1}$-RAD55*
NK6933<br>NK6934<br>NK6935	*MATa ura3-52 trp1-289 leu2-3,112 bar1::LEU2 rad55:TRP1-P$_{GAL1}$-RAD55*	NK1 *rad55:TRP1-P$_{GAL1}$-RAD55*
NK7188<br>NK7189	*MATa-inc trp1-289 leu2::P$_{GAL1}$-HO-LEU2 ura3-52::KAN-HO site-URA3 rad55::TRP1-P$_{GAL1}$-RAD55 rad54::NAT*	NK6724 *rad54::NAT*
NK7190		NK6725 *rad54::NAT*
NK7200<br>NK7201	*MATa ura3-52 trp1-289 leu2-3,112 bar1::LEU2 rad55:TRP1-P$_{GAL1}$-RAD55 rad54::NAT*	NK6933 *rad54::NAT*
NK7202		NK6934 *rad54::NAT*
NK7204<br>NK7205	*MATa ura3-52 trp1-289 leu2-3,112 bar1::LEU2 rad55:TRP1-P$_{GAL1}$-RAD55 srs2::HYG*	NK6933 *srs2::HYG*
NK7206		NK6934 *srs2::HYG*
NK7208<br>NK7209	*MATa ura3-52 trp1-289 leu2-3,112 bar1::LEU2 rad55:TRP1-P$_{GAL1}$-RAD55 srs2::HYG rad54::NAT*	NK7204 *rad54::NAT*
NK7210<br>NK7211		NK7206 *rad54::NAT*
NK7291<br>NK7292<br>NK7293	*MATa-inc trp1-289 leu2::P$_{GAL1}$-HO-LEU2 ura3-52::KAN-HO site-URA3 rad55::TRP1-P$_{GAL1}$-RAD55 srs2::HYG*	NK6724 *srs2::HYG*
NK7295<br>NK7296<br>NK7297	*MATa-inc trp1-289 leu2::P$_{GAL1}$-HO-LEU2 ura3-52::KAN-HO site-URA3 rad55::TRP1-P$_{GAL1}$-RAD55 rad54::NAT srs2::HYG*	NK7188 *srs2::HYG*
NK7424<br>NK7425<br>NK7426<br>NK7427<br>NK7428	*MATa-inc trp1-289 leu2::P$_{GAL1}$-HO-LEU2 ura3-52::KAN-HO site-URA3 rad55::TRP1-P$_{GAL1}$-RAD55 srs2::HYG*	NK6726 *srs2::HYG*
NK7764<br>NK7765	*MATa-inc trp1-289 leu2::P$_{GAL1}$-HO-LEU2 ura3-52::KAN-HO site-URA3 srs2::HYG rad51::TRP1 rad54::NAT*	NK5854 *rad51::TRP1 rad54::NAT*
NK7767<br>NK7768		NK5856 *rad51::TRP1 rad54::NAT*
NK9134	*MATa ura3-52 trp1-289 leu2-3,112 bar1::LEU2 rad55:TRP1-P$_{GAL1}$-RAD55 srs2::HYG rad54::NAT rad53::rad53-13Myc*	NK7208 *rad53::rad53-13Myc*
NK9135		NK7210 *rad53::rad53-13Myc*
NK10689<br>NK10690	*MATa ura3-52 trp1-289 leu2-3,112 bar1::LEU2 rad55:TRP1-P$_{GAL1}$-RAD55 htb2::htb2-mCherry-KAN nop56::nop56-CFP-URA3*	NK6933 *htb2::htb2-mCherry-KAN nop56::nop56-CFP-URA3*

NK10691		NK6934 *htb2::htb2-mCherry-KAN nop56::nop56-CFP-URA3*
NK10692<br>NK10693	*MATa ura3-52 trp1-289 leu2-3,112 bar1::LEU2 rad55:TRP1-*P_{GAL1}*-RAD55 srs2::HYG rad54::NAT htb2::htb2-mCherry-KAN nop56::nop56-CFP-URA3*	NK7208 *htb2::htb2-mCherry-KAN nop56::nop56-CFP-URA3*
NK10694		NK7210 *htb2::htb2-mCherry-KAN nop56::nop56-CFP-URA3*

Table S2. Oligonucleotides used in the study

Oligonucleotide number	Sequence (5′ to 3′)	Purpose
**OSM189**	ACGCCAGAAAATGTTGGTGATGCGCTT	To make *ARS1* probe
**OSM190**	ATCCACATCAATGGCTAATGGCAAAACT	To make *ARS1* probe
**OSM1006**	TGACTGGTACTACCGTAACGGTTC	qPCR at *ARO1* locus
**OSM1007**	GAATACCATCTGGTAATTCTGTAGTTTTGAC	qPCR at *ARO1* locus
**OSM2161**	TGTGGATATCTTGACTGATTTTTCC	To make *URA3* probe
**OSM2162**	ATACATGCATTTACTTATAATACAG	To make *URA3* probe
**OSM2233**	TGTATACTAAACTCACAAATTAGAGC	To monitor non-homologous DNA end cleavage with qPCR
**OSM2234**	CAACACTCAACCCTATCTCG	To monitor non-homologous DNA end cleavage with qPCR

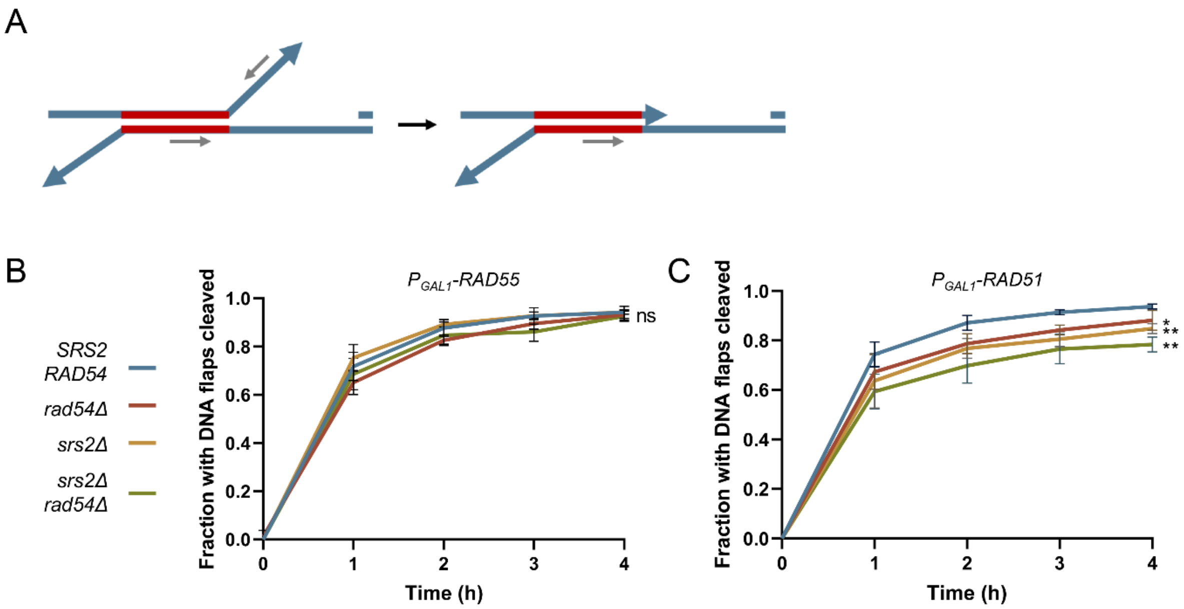

Figure S1. Analysis of non-homologous end cleavage during SSA. (**A**) A schematic representation of the annealing sites of the primers used to monitor the cleavage of the non-homologous DNA ends in the SSA system used in this work. The schematics on the left and right show the DNA structure before and after the non-homologous end cleavage respectively. Solid blue lines represent DNA strands in general, while solid red lines indicate homologous sequences used for the SSA repair. Grey arrows represent the primers used for the qPCR analysis. The PCR product can be generated only on the DNA prior to the end cleavage (left). (**B**) The analysis of non-homologous DNA end cleavage in the *Pgal1-RAD55* experiment (Figure 1F) using quantitative PCR. Fractions of the cells with non-homologous DNA ends cleaved are plotted over the duration of the time-course experiment. Asterisks describe the statistical significance of the difference between the value for the *Pgal1-RAD55* control and the values for each mutant derivative at the time point 4 h. The average ±SD of at least three biological repeats is shown for each time point of each genotype. Strains used: NK6724-NK6726; NK7188-NK7190; NK7291-NK7293; NK7295-NK7297. (**C**) The analysis of non-homologous DNA end cleavage in the *Pgal1-RAD51* experiment (Figure 1G) using quantitative PCR. Same colour-coding was used as in panel B. Fractions of the cells with non-homologous DNA ends cleaved are plotted over the duration of the time-course experiment. Asterisks describe the statistical significance of the difference between the value for *Pgal1-RAD51* control and the values for each mutant derivative at the time point 4 h. The average ±SD of at least three biological repeats is shown for each time point of each genotype. The p values of the two-sample Student's t-test are presented as follows: ns ($p > 0.05$), * ($p \leq 0.05$), ** ($p \leq 0.01$), *** ($p \leq 0.001$). Strains used: NK5858-NK5860; NK5861-NK5863; NK5864-NK5866; NK5868-NK5870.

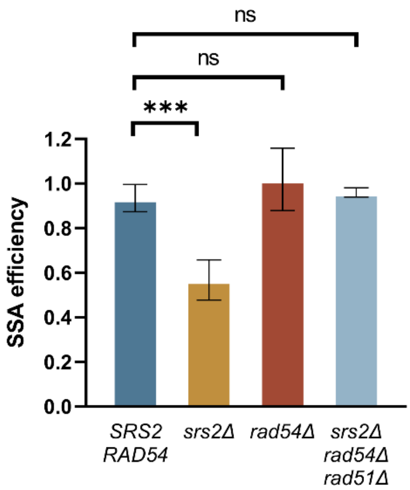

Figure S2. *RAD51* deletion fully suppresses the SSA defect in *srs2Δ rad54Δ* cells as determined by the plating assay. Average ±SD of at least three biological repeats is shown for each genotype. The p values of the two-sample Student's t-test are presented as follows: ns ($p > 0.05$), * ($p \leq 0.05$), ** ($p \leq 0.01$), *** ($p \leq 0.001$). Strains used: NK4691-NK4695; NK4805-NK4808, NK5854-NK5857; NK6390-NK6392; NK7764-NK7768.

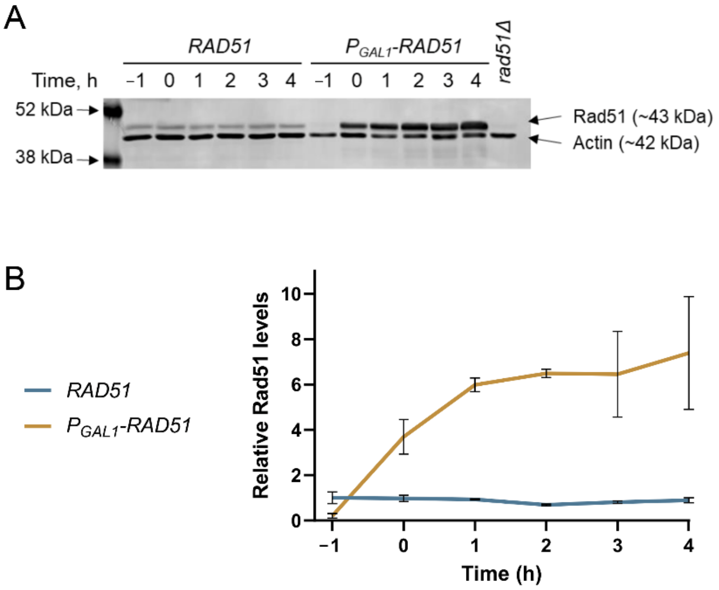

Figure S3. *RAD51* overexpression dynamics in the P_{GAL1}-*RAD51* background. (**A**) A representative image of the western blotting analysis performed to estimate the levels of Rad51 overproduction in the P_{GAL1}-*RAD51* cells upon the addition of galactose to the growth media. Samples were collected through a time-course experiment similar to the one shown in Figure 1G. (**B**) Quantification of the Rad51 levels through the time-course experiment in the cells with *RAD51* expressed from either P_{GAL1} or the endogenous P_{RAD51} promoter. The relative Rad51 levels were normalised against actin and then to the average value observed in the *RAD51* control at the timepoint -1 hour. The average ±SD of at least three biological repeats is shown for each time point of each genotype. Strains used: NK4691-NK4693; NK5858-NK5860.

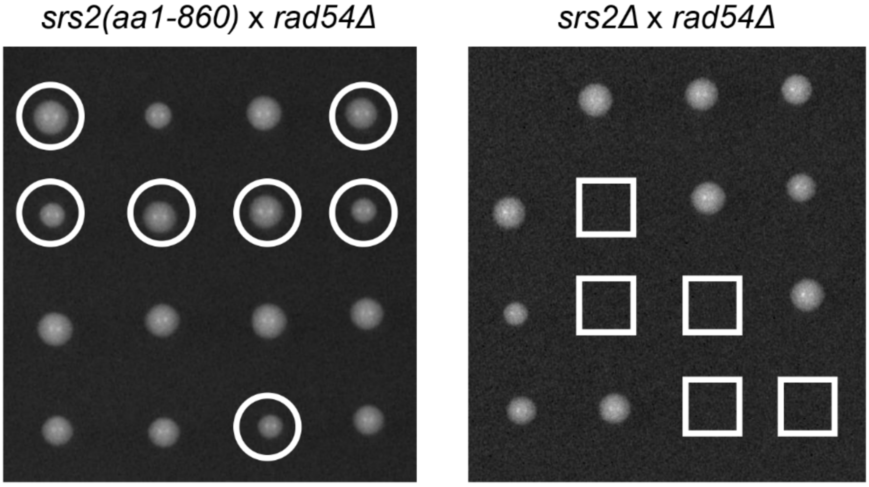

Figure S4. The mutant *srs2* allele that produces Srs2(aa1-860) protein lacking PIP, SIM and Rad51-interacting domains is not synthetically lethal with *rad54Δ*. Representative images of the tetrad dissections are shown for both diploid genotypes. White circles and squares indicate the *srs2(aa1-860) rad54Δ* and *srs2Δ rad54Δ* combinations, respectively. Each set of four colonies in a column originate from 4 spores of a single tetrad. Three biological repeats were dissected for the *srs2(aa1-860) rad54Δ* genotype and one for the *srs2Δ rad54Δ* control. Strains used: NK5901, NK5902, NK5903, NK5953.
